# Impact of the Dementia Care in Hospitals Program on acute hospital staff satisfaction

**DOI:** 10.1186/s12913-019-4489-z

**Published:** 2019-09-18

**Authors:** Margaret E. Murray, Anna Wong Shee, Emma West, Michelle Morvell, Meredith Theobald, Vincent Versace, Mark Yates

**Affiliations:** 1 Deakin University, Geelong Australia, Deakin Rural Health, School of Medicine, Warrnambool, VIC 3280 Australia; 2 Ballarat Health Services (BHS), Ballarat, 3350 Australia; 3 Deakin University, Geelong Australia, Faculty of Health, School of Medicine, Geelong, 3220 Australia

**Keywords:** Cognitive impairment, Staff satisfaction, Education, Acute hospital

## Abstract

**Background:**

To evaluate the impact of the Dementia Care in Hospitals Program (DCHP) on clinical and non-clinical staff job satisfaction, level of confidence and comfort in caring for patients with cognitive impairment (CI). Staff perceptions of how organisational support and hospital environment met the needs of patients with CI were also assessed.

**Methods:**

The DCHP was implemented across four acute hospital sites across Australia. Clinical and non-clinical staff received training on CI screening and communication strategies for patients with CI. A staff satisfaction survey was administered pre- and post-implementation of the DCHP.

**Results:**

One thousand seven hundred forty-eight staff received DCHP education and 1375 staff participated in the survey. Self-reported confidence and level of comfort in caring for patients with CI significantly improved following implementation. Staff also reported increased job satisfaction and organisational support at all hospital sites.

**Conclusions:**

The DCHP implementation within an acute hospital setting was found to show an improvement in staff confidence, comfort, and job satisfaction when caring for patients with CI. This study has significant implications for the improvement of care for patients with CI as well as staff retention and job satisfaction. Further research is required to determine whether these improvements are sustained in the longer term.

**Electronic supplementary material:**

The online version of this article (10.1186/s12913-019-4489-z) contains supplementary material, which is available to authorized users.

## Background

Acute hospitals are increasingly being required to provide care for people with cognitive impairment (CI). Over 83,000 people with CI are admitted to hospital each year in Australia [1]. Approaching 40% of all patients over the age of 65 admitted to hospital have been shown to have a CI, yet acute hospitals are not equipped to meet the needs of these patients [2]. Cognitive impairment is associated with a person’s decreased ability to think, formulate ideas, reason and remember, and includes dementia, delirium and other memory and thinking difficulties [3]. Patients with CI in acute hospitals are at a significantly increased risk of preventable complications such as falls, delirium, as well as adverse outcomes such as unexpected death, and unplanned entry into residential care [4]. The acute hospital environment can often be unfamiliar and distressing to patients with CI [4]. The National Safety and Quality Health Service Standards emphasise the importance of a hospital culture that provides safe and high-quality care tailored to the needs of patients with CI [5].

Staff awareness of CI, and the ability to identify and respond to patients with CI is fundamental to meeting the care and support needs of these patients and their carers [5]. Unlike other medical conditions, such as a fracture or a stroke, CI carries no visual stigmata, making it difficult to identify upon first contact by hospital staff. Furthermore, the diagnosis of CI within acute care can often be confounded by other illnesses, such as delirium [5]. Previous research has identified gaps in staff identification of patients with CI within the acute hospital environment [6]. There is a need to upskill acute hospital staff to identify patients with CI, which is the crucial first step in the provision of appropriate care and minimising patients’ risk of harm [5].

Previous research has shown that when hospital staff lack the appropriate knowledge and skills for the management of patients with CI, this results in low levels of confidence in their ability to care for these patients [7]. Staff require specific knowledge and skills to be able to respond appropriately to patients with CI. Best practice management and care of patients with CI includes: the identification and management of clinical risks, such as falls; targeted, individualised care; engagement with carers; appropriate management of behaviour; and delirium management and prevention strategies [8]. In addition, health professionals may feel uncomfortable discussing sensitive topics such as a person’s cognitive status, and avoid the topic, resulting in less than optimal care [9]. Communication skills training is well recognised as key strategy for improving staff confidence when caring for patients with CI [10]. The growing numbers of patients with CI in acute hospitals highlights the need for staff in acute hospital settings to be trained to respond to patients with CI appropriately [4].

Creating a culture which provides high-quality care to patients with CI, requires an all-of-hospital approach, where quality care is not solely the domain of nursing staff or health professionals. The ability of clinical and non-clinical staff to engage with patients with CI is critical for the provision of appropriate patient care and staff job satisfaction [11, 12]. Hospital staff have frequently reported difficulty engaging with patients with CI and their families [13] and caring for patients with CI is frequently linked to low job satisfaction and burnout in care staff [14]. The provision of staff training and education have been identified as important strategies in enhancing the psychological well-being and job satisfaction of staff [15]. A clinical and non-clinical staff approach to education is essential for job satisfaction amongst staff working with patients with CI and for the delivery of high-quality, consistent care for these patients and their carers.

### Aim

The aim of this study was to evaluate the impact of the Dementia Care in Hospitals Program (DCHP), a clinical and non-clinical staff education program designed to improve awareness of, and communication with patients with CI, on staff confidence, comfort and job satisfaction in caring for patients with CI.

## Methods

### Hospital sites

Four hospital sites located in South Australia, the Australian Capital Territory, Western Australia, and Tasmania, participants representing one regional and three metropolitan health services. All hospitals were tertiary hospitals with university affiliations, and the wards included were medical, surgical and aged care.

### Sample

Clinical (nursing, medical, allied health, pathology, radiology,) and non-clinical staff (food and domestic services, porters/ orderlies, security, cleaning and hotel services, engineering, volunteers, administration, gardeners, and maintenance) that were employed at each of the four hospital sites, were invited to participate in the DCHP educational training.

### Intervention

The intervention involved the implementation of the DCHP educational training program [16]. This program included:

1. Screening for CI of all patients aged 65 years and older or 50 years and over for an Aboriginal or Torres Strait Islander patients, using a validated screening tool [17].
2. Use of a Cognitive Impairment Identifier (CII) placed above the patient’s bedside to alert staff of patients with CI (See Additional file 1).
3. Employment of a set of nine key communication strategies by all staff (clinical and non-clinical) who engaged with the patients (See Additional file 2).

Hospital training was based on a “train the trainer” approach [18] and delivered by the DCHP team. The hospitals then commenced an implementation of this training to all relevant clinical and non-clinical staff, with direct patient contact.

The DCHP was implemented at each of the four hospital sites over a nine-month period between: December 2015 to April 2017.

### Staff satisfaction survey

Staff from each participating hospital site were invited to complete a staff satisfaction survey prior to and after implementation of the DCHP educational training program. The survey was developed in conjunction with Australian Institute for Primary Care and based on the literature and expert reviews. This survey consisted of five questions rated on a 5-point Likert scale, and three open-ended questions (See Additional files 3 and 4). It was designed to examine staff job satisfaction, as well as staff confidence and comfort in providing care for patients with CI. Staff perceptions of organisational support and how well equipped the hospital environment was to meet the needs of patients with CI, were also assessed.

### Data collection

Data collection commenced with the administration of the pre-intervention staff satisfaction ‘baseline’ survey. Commencement dates for the implementation of the DCHP were staged and the post-intervention survey was administered to staff at approximately 6 months post-intervention, to allow for the embedding of the DCHP at each hospital site. Intervention surveys were administered at the beginning of the DCHP training sessions in the hospitals. Post-intervention surveys were collected on the wards following implementation of the DCHP.

### Data analysis

Descriptive statistics including frequencies, means, and standard deviations were used to summarise the data. Independent *t-*tests were used to determine differences in pre- and post- DCHP education survey scores. All data were analysed using IBM SPSS 23.

## Results

### Participant demographics

A total of 1748 staff members (67.5%) of the total workforce staff participated in the educational sessions. Nursing staff accounting for the highest proportion of staff trained (*n* = 909, 52.0%), followed by non-clinical (*n* = 315, 18.0%), medical (*n* = 245, 14.0%), allied health (*n* = 210, 12.0%) and 69 (3.9%) staff did not identify their role. Prior to the DCHP intervention, 159 (89.3%) of the non-clinical staff had not previously received in-service or education on CI.

**Fig. 1:**
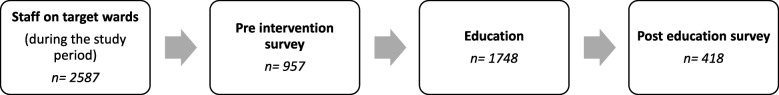
Participant numbers at each stage of implementation of the DCHP

The numbers of staff that completed training and completed surveys pre- and post-implementation are shown in Fig. 1.

### Staff satisfaction survey

#### Staff attitude

Prior to the intervention, the level of confidence reported amongst hospital staff was found to be highest for clinical staff, compared to non-clinical staff, with a significant improvement post-intervention (*p* < 0.001). The level of confidence reported amongst non-clinical staff also revealed a significant improvement post-intervention (*p* < 0.001). A similar significant finding was found for both clinical (*p* < 0.001) and non-clinical hospital staff (*p* < 0.001) who showed significant increases in level of comfort scores following the intervention phase. Job satisfaction also showed an overall increase across both clinical (*p* < 0.001) and non-clinical staff (*p* < 0.001) after the DCHP intervention (Table 1).

**Table 1:** Staff self-rated confidence, comfort and job satisfaction: pre and post-intervention

Self-rated measures	Pre-Intervention		Post-Intervention		t	*P*
	n	Mean (SD)	n	Mean (SD)		
Confidence						
Clinical staff	725	3.23 (0.73)	318	3.52 (0.80)	−5.66	< 0.001**
Non clinical staff	150	2.79 (0.93)	26	3.69 (0.79)	−4.65	< 0.001**
All staff	954	3.15 (0.79)	418	3.52 (0.79)	−7.98	< 0.001**
Comfort						
Clinical staff	724	3.24 (0.80)	317	3.55 (0.80)	−5.71	< 0.001**
Non clinical staff	150	2.92 (0.87)	26	3.54 (0.81)	−3.38	< 0.001**
All staff	953	3.18 (0.82)	417	3.53 (0.81)	−7.33	< 0.001**
Job Satisfaction						
Clinical staff	719	2.88 (0.77)	316	3.08 (0.79)	−3.75	< 0.001**
Non clinical staff	139	2.92 (0.80)	26	3.54 (0.86)	−3.57	< 0.001**
All staff	936	2.88 (0.79)	415	3.11 (0.82)	−4.70	< 0.001**

** Significant at the 0.001 level

Following the intervention, staff confidence and comfort increased significantly across all four hospital sites. Post-intervention, job satisfaction was also found to have increased significantly for Site 1 (*p* = 0.001) and Site 2 (*p* = 0.002). Although Site 3 and Site 4 reported a similar increase in job satisfaction, these changes were not statistically significant (Table 2).

**Table 2:** Staff self-rated confidence, comfort and job satisfaction at each hospital site: pre and post-intervention

Self-rated measures	Pre-Intervention		Post-Intervention		t	*P*
	n	Mean (SD)	n	Mean (SD)		
Confidence						
Site 1	234	3.24 (0.74)	40	3.73 (0.78)	−3.75	<.001**
Site 2	305	2.99 (0.86)	160	3.48 (0.78)	−5.93	<.001**
Site 3	237	3.24 (0.78)	137	3.56 (0.74)	−3.98	<.001**
Site 4	178	3.19 (0.70)	81	3.44 (0.88)	−2.48	0.02*
Comfort						
Site 1	234	3.15 (0.75)	40	3.75 (0.78)	−4.63	<.001**
Site 2	305	3.09 (0.87)	160	3.44 (0.82)	−4.15	<.001**
Site 3	237	3.28 (0.81)	136	3.60 (0.75)	−3.83	<.001**
Site 4	177	3.21 (0.83)	81	3.47 (0.87)	−2.25	0.03*
Job Satisfaction						
Site 1	232	2.83 (0.77)	40	3.30 (0.79)	−3.52	<.001**
Site 2	294	2.84 (0.79)	157	3.10 (0.83)	−3.16	0.002*
Site 3	234	3.00 (0.82)	137	3.12 (0.77)	−1.35	0.18
Site 4	176	2.87 (0.75)	81	3.01 (0.89)	−1.34	0.18

*Significant at the 0.05 level

** Significant at the 0.001 level

#### Organisational support and hospital environment

Prior to the intervention, staff-perceived level of organisational support for caring patients with CI, was found to be highest for clinical staff. Clincial staff perceptions of organisation support for patients with CI significantly improved post-intervention (*p* < 0.001) (Table 3). In contrast, staff perceptions of how well equipped the hospital environment was to meet the needs of patients with CI, was higher for non-clinical staff.

**Table 3:** Staff self-rated organisational support and hospital environment: pre and post-intervention

Self-rated measures	Pre-Intervention		Post-Intervention		t	*P*
	n	Mean (SD)	n	Mean (SD)		
Organisational Support						
Clinical staff	722	2.86 (0.81)	318	3.20 (0.90)	−5.99	< 0.001**
Non clinical staff	144	2.58 (0.96)	26	3.62 (0.85)	−5.11	< 0.001**
All staff	944	2.80 (0.85)	418	3.20 (0.91)	−7.84	< 0.001**
Hospital Environment						
Clinical staff	720	2.55 (0.81)	317	2.69 (0.88)	−2.53	0.010*
Non clinical staff	142	2.80 (0.96)	26	3.46 (0.76)	−3.33	0.001**
All staff	939	2.60 (0.86)	415	2.74 (0.90)	−2.75	0.010*

*Significant at the 0.05 level

** Significant at the 0.001 level

Post-intervention, staff perceptions of organisational support significantly increased for all of the four hospital sites (Table 4). Similarly, staff perceptions of how well equipped the hospital environment was in meeting the needs of patients with CI significantly increased post-intervention at Site 4 (*p* < 0.001).

**Table 4:** Staff self-rated organisational support and hospital environment at each hospital site: pre and post-intervention

Self-rated measures	Pre-Intervention		Post-Intervention		t	*P*
	n	Mean (SD)	n	Mean (SD)		
Organisational Support						
Site 1	233	2.70 (0.77)	40	3.03 (0.97)	−2.33	0.020*
Site 2	301	2.69 (0.84)	160	2.97 (0.95)	−3.20	0.001**
Site 3	233	2.97 (0.96)	137	3.42 (0.78)	−4.57	< 0.001**
Site 4	177	2.88 (0.79)	81	3.38 (0.90)	−4.58	< 0.001**
Hospital Environment						
Site 1	234	2.53 (0.80)	40	2.53 (0.93)	0.01	0.996
Site 2	295	2.52 (0.86)	157	2.66 (0.94)	−1.56	0.120
Site 3	233	2.95 (0.87)	137	2.88 (0.83)	0.79	0.430
Site 4	177	2.37 (0.77)	81	2.78 (0.91)	−3.76	< 0.001**

*Significant at the 0.05 level

** Significant at the 0.001 level

#### Staff engagement with patients with cognitive impairment and their carers

Clinical staff were more likely to report a problem or difficulty in working with patients and/or their carers compared to non-clinical staff. Prior to the DCHP intervention, (*n* = 722, 89.6%) clinical staff reported a problem or difficulty in working with patients with CI, and approximately two-thirds reported a problem or difficulty in working with their carers and/or family. Following the intervention, clinical staff showed a statistically significant decrease in the percentage staff experiencing difficulties working with patients with CI and their carers (Table 5).

**Table 5:** Staff reporting difficulty when working with patients and carers: by clinical status

Self-rated measures	Pre-Intervention		Post-Intervention		*P*
	n	%	n	%	
Difficulty working with patients with dementia, delirium or memory and thinking difficulties					
Clinical staff	722	89.6	318	80.6	0.001**
Non clinical staff	144	28.4	26	46.7	0.220
All staff	944	82.2	418	78.5	0.220
Difficulty working with the carer or family of patients with dementia, delirium or memory and thinking difficulties					
Clinical staff	720	65.9	317	49.1	< 0.001**
Non clinical staff	142	15.4	26	7.1	0.680
All staff	939	58.4	415	47.8	0.004*

*Significant at the 0.05 level

** Significant at the 0.001 level

## Discussion

This study is the first evaluation of the impact of the clinical and non-clinical staff education program on staff confidence, comfort and job satisfaction in caring for patients with CI. Overall, staff reported an increased ability to communicate with and respond appropriately towards patients with CI, following the implementation of the DCHP educational training program. Importantly, staff reported improved job satisfaction when working with patients with CI and their carers. Our findings show that the DCHP is effective in improving staff confidence, comfort, and job satisfaction when working with patients with CI.

Hospital staff require the necessary skills and knowledge to provide quality care to patients with CI, including how to identify, communicate and respond appropriately to patients with CI [5]. To the best of our knowledge, this study is the first to include non-clinical hospital staff, as these staff play an important role in the daily provision of care for patients with CI, and also require specialised CI training. Our findings show that the self-reported staff confidence and level of comfort in caring for patients with CI improved, following the implementation of the DCHP, for both clinical and non-clinical staff. These findings are consistent with a previous UK study conducted by Hughes et al. [19], which examined the knowledge and confidence of nursing and care assistant staff, in caring for people with dementia. It was found that although staff possessed adequate knowledge of dementia, staff confidence was lower, and could be positively influenced by additional training [19]. Similar to these findings, Sampson et al. [20], implemented a ‘train-the-trainer’ model across eight acute hospitals in the UK, and found that both clinical and non-clinical staff appeared more confident at engaging and responding to non-verbal cues in people with dementia. Utilising a clinical and non-clinical staff approach to education, is an important factor to increasing confidence for both clinical and non-clinical hospital staff, when working with patients with CI and their carers.

Staff-perceived difficulties when working with patients with CI, such as challenges communicating with patients, a lack of staff skills and understanding of CI, disruptive behaviours, and inadequate hospital resources, have been widely reported [13]. In this study, clinical staff reported experiencing less difficulty working with patients with CI and their carers following implementation of the DCHP. This is consistent with the findings of Surr et al. [15], who evaluated a specialist training programme for acute clinical hospital staff, in regards to staff attitudes towards the provision of care for people with dementia. That study showed that following the training programme, a significant positive change in staff attitudes was reported. In contrast, this current study found the perceived difficulty working with patients with CI to have increased amongst non-clinical staff, following DCHP implementation. The differences in findings between clinical and non-clinical staff may result from the varying degree of contact these two staff groups have with patients and their carers. Consistent with a higher level of patient contact amongst clinical staff, changes in their experiences with patients with CI and their carers, may also be easier to identify. Increasing staff skills in identifying and responding to patients with CI allows staff to deliver appropriate care and engage with these patients and their carers.

The ability of staff to engage with patients with CI and their carers can directly impact on staff job satisfaction [13]. Our study shows that overall, the DCHP improved staff perceived level of job satisfaction in working with patients with CI. These findings for clinical staff are consistent with previous research, where the level of knowledge in regards to CI, contributed significantly to job satisfaction of hospital employees [15]. That study showed that a specialist training programme significantly improved job satisfaction for clinical staff working with people with dementia in an acute hospital. While all of the hospital sites showed an improvement in job satisfaction, two of the sites did not demonstrate significant improvements. This may have been the result of variations in the implementation of the DCHP between hospital sites. Hospital sites were provided with the initial training by the DCHP team, however the implementation of the DCHP was site specific based on their hospital population, organisational support and resources. Our study also demonstrated an improvement in non-clinical staff-perceived level of job satisfaction in working with patients with CI. These findings are important as both clinical and non-clinical staff job satisfaction is positively related to patient satisfaction and the quality of care provided in an acute hospital setting [14, 21].

Within the hospital environment, the provision of high-quality care for patients with CI requires organisational support. While the involvement of clinical and non-clinical staff is important for driving cultural change, high-level organisational commitment and support is essential for sustaining and supporting this change [22]. In particular, hospitals with a negative culture of care, labelling people with dementia as ‘difficult’ have been found to negatively influence the well-being of people with dementia [23, 24]. Following the implementation of the DCHP, staff perceptions of the organisational support they received from the hospital improved overall. This is consistent with the literature which has highlighted the significant impact that the organisational and psychosocial working conditions, have on the health and well-being of people with CI [25]. Edvardsson et al. [25] provided recommendations for modifying an acute hospital environment, in order to better meet the needs of the older patients with CI. It was suggested that, adjusting the hospital environment by providing a balanced approach to care, which includes the provision of core knowledge and skills for all staff, and access to CI expertise in acute hospitals. This suggests that in order for staff training to affect the acute care outcomes for patients with CI, organisational change within the hospital environment is necessary [26].

### Limitations

One of the limitations of this study was that the fidelity of the DCHP education implementation was not assessed and may have impacted the outcomes. However, this is a pragmatic study and involved implementing the DCHP education within the constraints of a real clinical environment, respecting that differences existed between the participating hospitals as a result of their hospitalised population, practice differences, data coding differences and organisational resources. An additional limitation is that the sustainability of the education program was not measured. However, this is an important measure to examine and should be the basis of further research. The post-intervention survey responses were lower than the pre-intervention survey responses. This was because the pre-intervention survey was distributed during planned DCHP training sessions in the hospitals. The post-intervention surveys were distributed to staff on the wards, up to 6 months post intervention, and it is likely that the timing and distribution method impacted on the response rate. Psychometrics were not completed for the survey used in this study and this is an acknowledged limitation.

## Conclusions

Overall, staff reported increased confidence, comfort and job satisfaction in caring for patients with CI, following the implementation of the DCHP education program. The overall positive results around staff satisfaction have significant implications for the improvement of care for patients with CI, as well as staff retention and job satisfaction. Further research is required in order to determine whether these improvements are sustained in the long term.

## Additional files

Additional file 1: Cognitive Impairment Identifier (CII). The CII bedside alert is a copyright product of Ballarat Health Services. Patients who screen positive for CI are offered placement of the CII above their bedside. The CII is a key component of the DCHP and its visibility enables all hospital staff (clinical and non-clinical) to assist patients with CI. (PDF 139 kb)

Additional file 2: Dementia Care in Hospitals Program Key Communication Strategies. The nine key communication strategies used as part of the DCHP educational training program. (PDF 137 kb)

Additional file 3: Pre-Intervention Staff Satisfaction Survey. The staff satisfaction survey completed by staff prior to implementation of the DCHP educational training program. (PDF 185 kb)

Additional file 4: Post-Intervention Staff Satisfaction Survey. The staff satisfaction survey completed by staff after implementation of the DCHP educational training program. (PDF 186 kb)

## Data Availability

The datasets used and analysed during the current study available from the corresponding author on reasonable request.

## Abbreviations

CI: Cognitive impairment
CII: Cognitive Impairment Identifier
DCHP: Dementia Care in Hospitals Program
