# Supplementary material for: Impact of the Dementia Care in Hospitals Program on acute hospital staff satisfaction
Source: BMC Health Serv Res. 2019 Sep 18;19:680. doi: 10.1186/s12913-019-4489-z (PMC6751685; doi:10.1186/s12913-019-4489-z)

Additional file 1: Cognitive Impairment Identifier (CII). The CII is a copyright product of Ballarat Health Services.

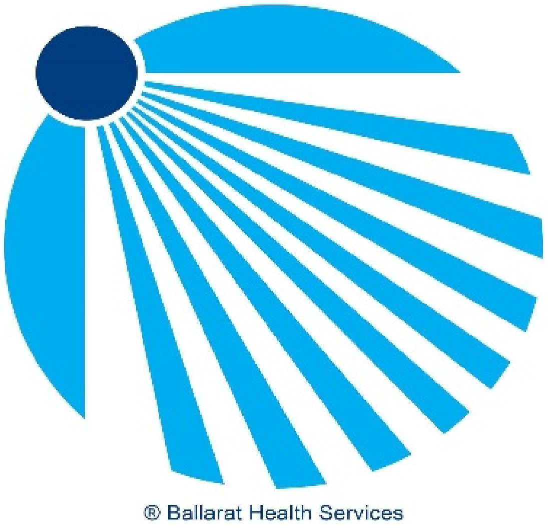

Supplement: Supplementary file 1 — Cognitive Impairment Identifier (CII). The CII bedside alert is a copyright product of Ballarat Health Services. Patients who screen positive for CI are offered placement of the CII above their bedside. The CII is a key component of the DCHP and its visibility enables all hospital staff (clinical and non-clinical) to assist patients with CI. (PDF 139 kb) [file 12913_2019_4489_MOESM1_ESM.pdf]
