# Supplementary material for: Impact of the Dementia Care in Hospitals Program on acute hospital staff satisfaction
Source: BMC Health Serv Res. 2019 Sep 18;19:680. doi: 10.1186/s12913-019-4489-z (PMC6751685; doi:10.1186/s12913-019-4489-z)
Supplement: Supplementary file 2 — Dementia Care in Hospitals Program Key Communication Strategies. The nine key communication strategies used as part of the DCHP educational training program. (PDF 137 kb) [file 12913_2019_4489_MOESM2_ESM.pdf]

Appendix B: Dementia Care in Hospitals Program Key Communication Strategies

Introduce yourself

- approach the patient from the front
- reduce distractions
- ensure the patient is using aids
- give your name and a brief explanation of who you are and why you are there

Involve carers

- identify relevant carers at admission
- involving carers helps to deliver quality care by providing knowledge and expertise
- carers may be unsure about how things work or whom they should to talk to if they have concerns
- improving communication with carers as advocates for patients promotes beneficial outcomes

Make eye contact

- gain the persons attention
- usual visual cues from patient and communicator
- be mindful that eye contact may communicate very different things to people of various cultures

Remain calm and talk in a matter of fact way

- think about how you are presenting yourself
- consider your facial expression, body language, mood, tone and pitch of voice
- your approach can set the tone of the interaction

Keep sentences short and simple

- speak slowly
- use familiar, common words
- express long or complex messages using several shorter, simpler sentences
- pause between sentences

Focus on one instruction at a time

- give one instruction
- allow time for processing
- provide physical prompts / visual supports
- don't bombard with additional requests or questions

Give time for a response

- be a patient listener
- wait for a response before continuing
- be an active, sensitive and creative listener

Repeat yourself… don't assume you've been understood

- wait for a reply or evidence of comprehension
- repeat or rephrase the message

Do not give too many choices

- use 'yes/no' or 'either/or' questions
- avoid open-ended question
