# Supplementary material for: Impact of the Dementia Care in Hospitals Program on acute hospital staff satisfaction
Source: BMC Health Serv Res. 2019 Sep 18;19:680. doi: 10.1186/s12913-019-4489-z (PMC6751685; doi:10.1186/s12913-019-4489-z)
Supplement: Supplementary file 4 — Post-Intervention Staff Satisfaction Survey. The staff satisfaction survey completed by staff after implementation of the DCHP educational training program. (PDF 186 kb) [file 12913_2019_4489_MOESM4_ESM.pdf]

Additional file 4: Post-Intervention Staff Satisfaction Survey

Dementia Care in Hospitals Program

Staff satisfaction survey – post education

Before you receive education about the Dementia Care in Hospitals Program, we are interested in your views and experience of dealing with patients with cognitive impairment and their carer/family. The information will assist the hospital in training staff, improving the quality of care for these patients, and improving communication with carers/families.

All replies will be strictly confidential and you will not be identified in any way.

Please tick the box which best describes your position.

☐ **clinical staff,** e.g. nursing, medical, allied health etc ☐ **non-clinical staff** e.g. engineers, ward clerks, etc

Non-clinical Staff: Have you ever been offered in-service or education on dementia or delirium?

Yes ☐ No ☐

1. What proportion of patients do you think you come across in the hospital with dementia, delirium or memory and thinking difficulties?

**10%**	**20%**	**30%**	**40%**	**50%**	**60%**	**70%**	**80%**	**90%**
☐	☐	☐	☐	☐	☐	☐	☐	☐

2. How would you rate your confidence in dealing with patients with dementia, delirium or memory and thinking difficulties?

very low ☐ low ☐ satisfactory ☐ high ☐ very high ☐

3. How would you rate your level of comfort in dealing with patients with dementia, delirium or memory and thinking difficulties?

very low ☐ low ☐ satisfactory ☐ high ☐ very high ☐

4. How would you rate the level of organisational support you receive when dealing with patients with dementia, delirium or memory and thinking difficulties?

very low ☐ low ☐ satisfactory ☐ high ☐ very high ☐

5. How would you rate your level of job satisfaction in dealing with patients with dementia, delirium or memory and thinking difficulties?

very low ☐ low ☐ satisfactory ☐ high ☐ very high ☐

6. In your experience how well equipped is the hospital environment to meet the needs of patients with dementia, delirium or memory and thinking difficulties?

very low ☐ low ☐ satisfactory ☐ high ☐ very high ☐

7. Have you experienced any problem or difficulty working with patients with dementia, delirium or memory and thinking difficulties?

Yes ☐ No ☐

If you answered yes, please list the 3 most significant difficulties:

1. ....

....

2. ....

....

3. ....

....

8. What do you think is important in working or communicating effectively with patients with dementia, delirium or memory and thinking difficulties? Please list the 3 most important:

1. ....

....

2. ....

....

3. ....

....

9. Have you experienced any problem or difficulty working with the carer or family of patients with dementia, delirium or memory and thinking difficulties?

Yes ☐ No ☐

If you answered yes, please list the 3 most significant difficulties:

1. ....

....

2. ....

3. ....
